# A Role for NADPH Oxidase in Antigen Presentation

**DOI:** 10.3389/fimmu.2013.00295

**Published:** 2013-09-23

**Authors:** Gail J. Gardiner, Sarah N. Deffit, Shawna McLetchie, Liliana Pérez, Crystal C. Walline, Janice S. Blum

**Affiliations:** ^1^Department of Microbiology and Immunology, Indiana University School of Medicine, Indianapolis, IN, USA

**Keywords:** NADPH oxidase, B lymphocytes, chronic granulomatous disease, autoimmunity, antigen presentation

## Abstract

The nicotinamide adenine dinucleotide phosphate (NADPH) oxidase expressed in phagocytes is a multi-subunit enzyme complex that generates superoxide (O_2_^.−^). This radical is an important precursor of hydrogen peroxide (H_2_O_2_) and other reactive oxygen species needed for microbicidal activity during innate immune responses. Inherited defects in NADPH oxidase give rise to chronic granulomatous disease (CGD), a primary immunodeficiency characterized by recurrent infections and granulomatous inflammation. Interestingly, CGD, CGD carrier status, and oxidase gene polymorphisms have all been associated with autoinflammatory and autoimmune disorders, suggesting a potential role for NADPH oxidase in regulating adaptive immune responses. Here, NADPH oxidase function in antigen processing and presentation is reviewed. NADPH oxidase influences dendritic cell (DC) crosspresentation by major histocompatibility complex class I molecules through regulation of the phagosomal microenvironment, while in B lymphocytes, NADPH oxidase alters epitope selection by major histocompatibility complex class II molecules.

## NADPH Oxidase

**Figure 1:**
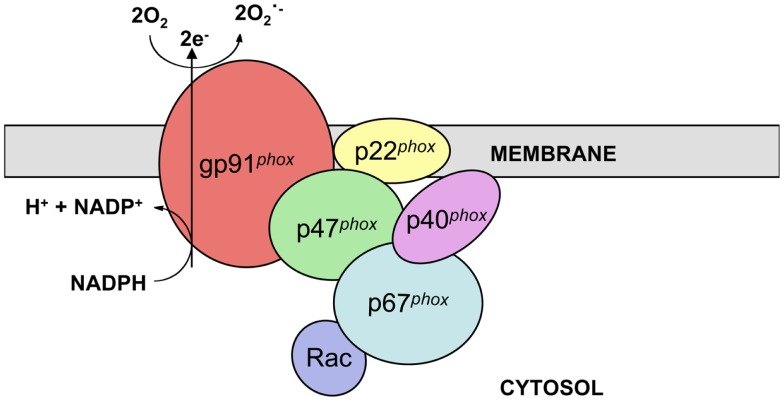
**NADPH oxidase structure**. NADPH oxidase is a multi-subunit enzyme complex present in the vesicular and plasma membranes of leukocytes. Two transmembrane subunits, gp91*^phox^* and p22*^phox^*, make up flavocytochrome *b_558_*, the catalytic core of the complex. This heterodimer catalyzes the transfer of electrons from cytosolic NADPH to molecular oxygen, thus generating superoxide. Flavocytochrome *b_558_* is regulated by association with the subunits p47*^phox^*, p67*^phox^*, and p40*^phox^* along with the small GTPase Rac. These regulatory subunits are present in the cytosol of resting cells and translocate to the catalytic core following stimulation.

The phagocyte nicotinamide adenine dinucleotide phosphate (NADPH) oxidase complex catalyzes electron transfer from NADPH to molecular oxygen and thus generates superoxide (O_2_^.−^), the precursor of H_2_O_2_ and other reactive oxygen species (ROS) necessary for the microbicidal activity of neutrophils and other phagocytes during innate immune responses. The active phagocyte oxidase (*phox*) complex consists of five subunits and the small GTPase Rac. The catalytic core of the complex, which displays little to no activity in resting phagocytic cells, is comprised of two transmembrane subunits, gp91*^phox^* (often referred to as NOX2) and p22*^phox^*. These proteins form a heterodimer known as flavocytochrome *b_558_* in vesicular and plasma membranes of neutrophils and other leukocytes. In response to stimuli, the regulatory subunits p47*^phox^*, p67*^phox^*, and p40*^phox^*, which together exist as a complex in the cytosol, translocate along with Rac-GTP to the membrane-bound catalytic core to form the active enzyme complex (Figure 1) (1–3).

## Chronic Granulomatous Disease

Inherited mutations in the subunits of NADPH oxidase result in chronic granulomatous disease (CGD), a primary immunodeficiency disorder affecting at least 1 in 250,000 individuals in the US (4). X-linked CGD, the most common genetic subgroup, is caused by mutations in gp91*^phox^*. X-linked CGD accounts for approximately 70% of CGD cases and affects mostly males, although female carriers with skewed X-chromosome inactivation can also display a CGD phenotype (4–7). Mutations in p22*^phox^*, p47*^phox^*, and p67*^phox^* can also give rise to CGD through autosomal recessive inheritance. Patients with autosomal recessive forms of CGD are often diagnosed later in life, exhibit a milder clinical phenotype, and live longer than patients with X-linked CGD (4, 7). Isolated cases of children with a CGD phenotype having mutations in Rac2 (8) and p40*^phox^* (9) have also been described.

Phagocytes lacking functional NADPH oxidase are unable to produce ROS necessary to kill certain pathogenic bacteria and fungi. Thus, patients with CGD experience recurrent infections, most often with catalase positive organisms. Patients are also prone to develop severe granulomas resulting in obstructive lesions in the esophagus, stomach, and urinary tract (4, 7, 10, 11). In a cohort of 368 CGD patients from the US, pneumonia was the most common form of infection (79% of patients with at least one episode), and 41% of these infections were caused by *Aspergillus* spp. Abscesses were also common (68% of patients with at least one episode). Additionally, suppurative adenitis, osteomyelitis, septicemia, cellulitis, and meningitis were reported (4). Similar data were described in a European cohort of 429 CGD patients (7). In both cohorts, the most common causes of death were infections involving *Aspergillus* spp. and *Burkholderia cepacia* (4, 7).

## CGD and Autoimmunity

Remarkably, CGD is also associated with a number of autoinflammatory and autoimmune disorders. Several patients with systemic lupus erythematosus (SLE) and discoid lupus erythematosus (DLE) have been described (4, 7, 12, 13). In addition, lupus has also been widely reported in female carriers of X-CGD (4, 7, 12–15). A number of other studies have pointed to an association of CGD with autoimmune arthritis (7, 15, 16). CGD patients with colitis-like symptoms (4, 9) similar to inflammatory bowel disease, myasthenia gravis (4), immune thrombocytopenia (4, 7), sarcoidosis (17), pericardial effusion, IgA nephropathy, and aphthous stomatitis (15) have also been described.

In addition to this well-established connection between CGD and autoimmunity, genes encoding oxidase subunits have been associated with autoinflammatory and autoimmune disorders in genome-wide association studies (GWAS). A variation in *NCF2*, the gene encoding p67*^phox^*, has been identified as an important risk factor for SLE (18). *NCF4*, encoding p40*^phox^*, has been associated with rheumatoid arthritis (19) and Crohn’s disease (20, 21). In the absence of NADPH oxidase activity, murine dendritic cells (DC) produce elevated levels of inflammatory cytokines that alter helper T cell differentiation and promote development of collagen-induced arthritis (22, 23). These findings suggest NADPH oxidase may be playing an important role not only in innate immune responses, but also in adaptive immune responses.

## NADPH Oxidase and Crosspresentation

Several studies have looked into the relationship between NADPH oxidase activity and antigen crosspresentation. DC, and to a lesser extent, macrophages, are capable of crosspresentation, a process by which peptides derived from exogenous antigens are loaded onto and presented by major histocompatibility complex class I (MHC-I) molecules. During crosspresentation, exogenous antigens, either particulate or soluble, are phagocytosed or endocytosed. These antigens are proteolytically processed to short peptides, 8–10 amino acids long, and subsequently loaded onto MHC-I. Crosspresentation has been implicated in infection responses where uninfected DC play a critical role in CD8^+^ T cell activation (24–26). Crosspresentation of self and environmental antigens may also play a role in the maintenance of central and peripheral tolerance, implicating crosspresentation in the development of autoimmune disorders and as a potential target for immunotherapy (27–30).

**Figure 2:**
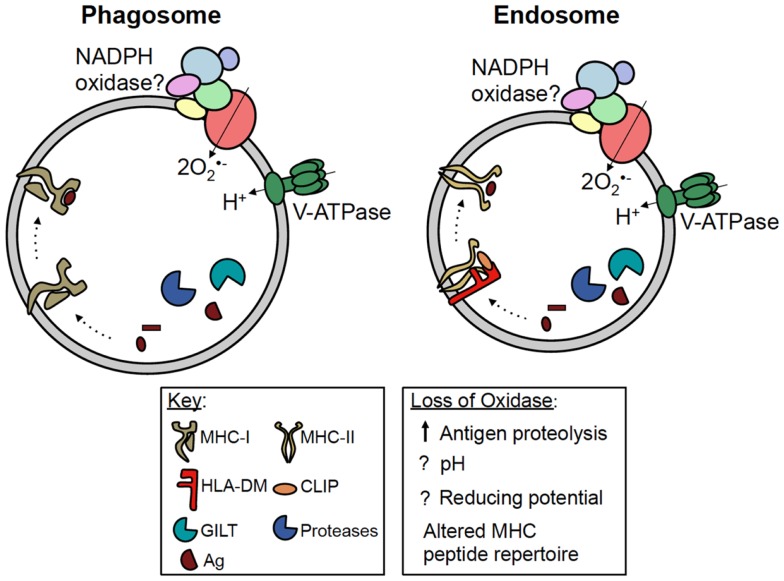
**Potential roles of NADPH oxidase in antigen presentation**. MHC-I and MHC-II are both present in phagosomes and endosomes of antigen presenting cells. For simplicity, MHC-I has been shown in a phagosome, while MHC-II has been shown in an endosome. During crosspresentation, exogenous antigens can be processed within these compartments by proteases, prior to loading onto MHC-I. NADPH oxidase has been shown to regulate antigen processing and MHC-I crosspresentation in DC; however, whether NADPH oxidase regulates this process by modulating the phagosomal pH or redox microenvironment is currently debated. During classical MHC-II presentation, exogenous antigens are processed within vesicular compartments by proteases and GILT. Following the removal of CLIP by HLA-DM, processed peptides are loaded onto MHC-II. NADPH oxidase can also regulate MHC-II presentation in B cells by altering the peptide repertoire displayed by MHC-II, possibly in favor of self antigens. However, the mechanism underlying this phenomenon is still unclear. Whether or not NADPH oxidase, MHC-I and -II, and the antigen processing machinery co-localize within the same vesicular compartment also remains to be elucidated.

The pathways by which exogenous antigens encounter MHC-I are incompletely understood. Some studies have shown crosspresentation is dependent on proteasome activity, suggesting exogenous antigens are transported into the cytosol for processing (31, 32). In DC, studies have shown phagosomes fuse with the endoplasmic reticulum (ER), and antigenic peptides processed in the cytosol are transported by TAP back into the phagosome lumen before being loaded onto MHC-I (33). Other studies indicate exogenous antigens are processed within phagosomes and endosomes prior to intersecting MHC-I, independent of the proteasome and TAP (Figure 2) (34, 35). In DC, Amigorena and colleagues identified a central role for NADPH oxidase in modulating the efficiency of antigen degradation and MHC-I crosspresentation to CD8^+^ T cells.

To investigate *in vivo* oxidase function in crosspresentation, wild type and gp91*^phox^*-deficient mice were immunized with ovalbumin coupled to an antibody specific for the receptor DEC205, targeting this antigen-antibody complex to murine DC. CD8^+^ T cells that recognize epitopes from ovalbumin in the context of MHC-I were adoptively transferred into recipient wild type or oxidase-deficient mice, followed by an analysis of T cell activation (36). Ovalbumin-specific T cell activation was significantly less efficient in gp91*^phox^*-deficient mice compared to wild type mice. Results *in vitro* also showed gp91*^phox^*-deficient DC did not efficiently crosspresent ovalbumin to antigen-specific CD8^+^ T cells compared to wild type DC. However, presentation of the pre-processed ovalbumin SIINFEKL peptide was similar in both DC populations. Together, these data suggest gp91*^phox^* is important for antigen processing and crosspresentation in murine DC (36).

Similarly, *in vitro* human CD8^+^ T cell recognition of an HLA-A2-restricted extended MelanA/MART-1 peptide was reduced in DC from CGD patients and in DC treated with an oxidase inhibitor compared to wild type DC, whereas presentation of a shorter form of this peptide was not affected (37). Chemical inhibition of the oxidase did not alter DC phagocytosis. Given that crosspresentation of the long, but not short, MelanA/MART-1 peptide requires processing by DC, these results indicate an important role for NADPH oxidase in antigen processing and crosspresentation by human DC (37).

Amigorena and colleagues found this effect of NADPH oxidase on crosspresentation occurs in murine CD8^+^ but not CD8^−^ DC. Rac2, the small GTPase which facilitates oxidase subunit assembly on phagosomal membranes, was also required for efficient crosspresentation (38).

Precisely how loss of oxidase function disrupts MHC-I crosspresentation remains controversial. Amigorena and colleagues have proposed in DC that NADPH oxidase promotes an alkalinization of the phagosome lumen thus limiting antigen proteolysis and preserving epitopes for crosspresentation by MHC-I (36–39). FACS-based studies using pH sensitive fluorophores coupled to latex beads revealed pH values of 7.5 or higher after phagocytosis in phagosomes of DC with functional oxidase, while rapid acidification of phagosomes was observed in DC from gp91*^phox^*-deficient mice (36). Loss of ovalbumin on coated beads after phagocytosis suggested enhanced degradation of this antigen in gp91*^phox^*-deficient cells compared with wild type DC, consistent with a role for the oxidase in limiting antigen processing for crosspresentation (36).

Yates and colleagues also found in the absence of functional NADPH oxidase, the rate of phagosomal proteolysis in DC was significantly increased compared to cells with normal oxidase activity (40). However, results of excitation ratio fluorometry and real-time fluorometric confocal microscopy experiments suggested NADPH oxidase activity does not prevent acidification of DC phagosomes (40).

To determine an alternative mechanism by which NADPH oxidase activity limits antigen proteolysis, the effect of oxidase activity on different classes of proteases was examined. Phagosomal hydrolysis of fluorogenic substrates for cysteine cathepsins B/S/L and aspartic cathepsins D/E was monitored in oxidase-proficient and -deficient DC in real time. NADPH oxidase ROS production inhibited phagosomal cysteine, but not aspartic, cathepsin activity. This inhibition also occurred in the presence of H_2_O_2_ in a reconstituted system of lysosomal extracts and could be reversed upon addition of reduced glutathione, suggesting oxidase activity may limit antigen proteolysis through redox modulation of cysteine cathepsin activity. Using an assay with bead coupled cysteine-linked fluorochromes, disulfide bond reducing capacity was also found to be markedly inhibited within phagosomes of wild type DC compared to gp91*^phox^*-deficient DC, suggesting NADPH oxidase activity may also be important for regulating disulfide bond reduction in antigen processing events (40).

Discrepancies regarding the effect of NADPH oxidase activity on phagosomal pH in DC remain. However, all studies highlight a role for NADPH oxidase in limiting antigen proteolysis in phagosomes (36, 40). Whether the reduced antigen crosspresentation by DC outlined in these reports perturbs the development of self tolerance in CGD patients remains unclear.

## NADPH Oxidase and MHC-II Antigen Presentation

A new genetic subgroup of CGD was recently defined upon identification of a patient with autosomal recessive mutations in both copies of *NCF4*, the gene encoding p40*^phox^* (9). Familial studies to track p40*^phox^* mutations in this patient revealed the paternal allele encodes a truncated protein due to the presence of a premature stop codon. The maternal allele contains an R105Q missense mutation in the phospholipid binding PX (*phox* homology) domain. In neutrophils, this mutation results in a loss of p40*^phox^* phosphatidylinositol (3,4,5)-triphosphate binding followed by premature dissociation from the neutrophil phagosome and ultimately impaired production of intracellular ROS by the oxidase complex (9).

This patient did not initially present with recurrent pathogenic infections characteristic of CGD, but rather with severe granulomatous colitis (9). *NCF4* has been associated with ileal Crohn’s disease (20, 21) as well as rheumatoid arthritis (19) in GWAS. These linkages to autoinflammatory and autoimmune disorders suggest a potential role for p40*^phox^* in regulating adaptive immune responses. Supporting this hypothesis, human B lymphocytes lacking functional p40*^phox^* exhibit altered antigen presentation by major histocompatibility complex class II (MHC-II) molecules (41).

MHC-II molecules are heterodimeric transmembrane proteins composed of α and β subunits. Within professional antigen presenting cells, these αβ heterodimers are loaded with peptides derived from processed protein antigens prior to cell surface presentation to CD4^+^ T cells. MHC-II assemble in the ER where they bind invariant chain (Ii) (42). Ii stabilizes the MHC-II heterodimer and directs the trafficking of MHC-II through the Golgi and into the endosomal network (43). Within this network, Ii is systematically cleaved by acid proteases leaving class II-associated invariant chain peptide (CLIP) bound to the MHC-II binding groove (44–46). In endosomes, HLA-DM, a non-classical MHC-II protein, catalyzes the removal of CLIP, and further edits peptides that bind MHC-II to favor the presentation of stable antigenic epitopes (Figure 2) (47, 48).

Antigenic peptides capable of binding MHC-II are generated within the endosomal network by proteases active at acidic pH, including cathepsins and asparaginyl endopeptidase (AEP) (49). Antigen proteolysis can be influenced by gamma-interferon-induced lysosomal thiol reductase (GILT). This enzyme reduces antigen disulfides to favor protein unfolding at acidic pH (50, 51). Whether or not NADPH oxidase co-localizes with MHC-II, GILT, and acidic proteases in endosomes remains unclear (Figure 2).

Interestingly, evidence also suggests a role for ROS in regulating antigen presentation by MHC-II. In phagocytes, nitric oxide production is required for processing bacterial polysaccharides into fragments capable of binding MHC-II for presentation to T cells (52). Conflicting reports with B lymphocytes also suggest roles for superoxide and NADPH oxidase in MHC-II presentation, yet these early studies employed samples from reported CGD patients without genetic analysis to confirm defective oxidase subunits (53, 54). Although B lymphocytes express functional NADPH oxidase subunits (55), the precise function of this enzyme complex in these cells remains unclear. Analysis of CGD patient B cells (56, 57) revealed alterations in circulating memory B cells; however, inactivation of the oxidase also impacts T lymphocyte development and function (58–60).

To examine the role of the oxidase in cytoplasmic antigen presentation by MHC-II, p40*^phox^* expression in a human B cell line was disrupted using lentiviral-driven shRNA (41). In B lymphocytes, p40*^phox^* deficiency altered the profile of antigenic epitopes displayed by MHC-II, with a shift toward preferential presentation of self membrane-associated antigens. Studies using a panel of antigen-specific T cells revealed MHC-II presentation of epitopes from cytoplasmic and exogenous antigens was reduced with knockdown of p40*^phox^* in B cells. Yet, MHC-II presentation of epitopes from membrane antigens was enhanced for B cells deficient in p40*^phox^* when compared to parental or control cells with normal levels of this subunit. MHC-II presentation of synthetic short peptides by p40*^phox^*-deficient B cells was comparable to control cells, revealing that loss of this subunit did not influence peptide binding to surface MHC-II or T cell recognition of these complexes. Consistent with these results, surface levels of HLA-DR in parental and control shRNA-treated cells were equivalent (41). These results suggest that diminished p40*^phox^* expression may modulate the balance of foreign versus self antigen presented in the context of MHC-II in human B cells.

To determine whether spontaneously arising mutations in human p40*^phox^* influence MHC-II presentation, B lymphoblastoid cells were generated from the CGD patient with mutations in *NCF4* (9) and transduced to express the MHC-II allele DRβ1*0401 (61). These patient-derived cells were also transduced to express functional wild type p40*^phox^* or the mutant R105Q allele (41). In agreement with previous results, reduced CD4^+^ T cell responses were detected in response to exogenous antigens in patient B cells with mutated p40*^phox^* compared to patient cells reconstituted with wild type p40*^phox^*. MHC-II presentation of epitopes from endogenous, self membrane antigens was similar regardless of the form of p40*^phox^* expressed by these B cells. T cell responses to synthetic peptides presented in the context of MHC-II on patient-derived B cells were comparable for cells expressing mutant or wild type forms of p40*^phox^* (41).

Notably, presentation of exogenous tetanus toxoid was also reduced in B cells from CGD patients with mutations in gp91*^phox^* as well as B cells from the patient with mutations in p40*^phox^*. No differences were observed in the expression levels of key components of the MHC-II pathway including HLA-DR, -DM, or -DO in B cells from the patient with *NCF4* mutations. While reduced presentation of exogenous antigens was consistently observed with reduced oxidase p40*^phox^*, the ability of these B cells to endocytose antigen was similar to cells from healthy individuals. Flow cytometric analysis revealed patient-derived B cells deficient in p40*^phox^* spontaneously produced less intracellular superoxide compared to these CGD cells with restored wild type p40*^phox^* expression. Yet as observed in patient neutrophils (9), extracellular ROS production in response to PMA was similar for the patient and wild type p40*^phox^*-reconstituted B cells (41). Taken together, these data suggest the altered profile of epitopes selected for presentation in p40*^phox^*-deficient B cells may be linked to diminished intracellular superoxide production.

While p40*^phox^* is known to regulate oxidase activation in response to specific stimuli in neutrophils, this marks the first demonstration of a role for p40*^phox^* in antigen presentation and B cell functions. These results suggest functional p40*^phox^* expression can influence the peptide repertoire presented by MHC-II on B cells and the subsequent activation of CD4^+^ T cells. Thus, p40*^phox^* deficiency may contribute to autoimmune and autoinflammatory disease predisposition in patients with CGD or oxidase gene polymorphisms.

## Conclusion

Inherited defects in NADPH oxidase give rise to CGD, a primary immunodeficiency that has been linked with a number of autoinflammatory and autoimmune disorders. Genes encoding the subunits of NADPH oxidase have also been associated with these disorders in GWAS. Together, these observations suggest a potential role for NADPH oxidase in regulating adaptive immune responses. Currently, studies have shown NADPH oxidase activity limits antigen proteolysis in DC phagosomes to promote MHC-I crosspresentation; however, the mechanism underlying this effect remains unclear. Some studies suggest NADPH oxidase activity limits antigen proteolysis by preventing phagosome acidification, while others suggest it alters the local redox microenvironment within the phagosome. Studies from our laboratory have shown NADPH oxidase also modulates MHC-II presentation in B cells and that defects in oxidase activity may alter the peptide repertoire displayed by MHC-II. This phenomenon could contribute to the development of autoimmunity in patients with CGD or polymorphisms in oxidase genes. Future studies are needed to further establish this link as well as to elucidate the mechanism by which NADPH oxidase regulates antigen presentation.

## Author Contributions

Gail J. Gardiner performed the literature review of NADPH oxidase, CGD, connections to autoimmunity, NADPH oxidase and crosspresentation, and NADPH oxidase and MHC-II presentation and also wrote the manuscript. Sarah N. Deffit reviewed crosspresentation and MHC-II presentation and wrote these sections of the manuscript. Shawna McLetchie and Liliana Pérez designed the figures and wrote the figure legends. Crystal C. Walline reviewed CGD connections to autoimmunity and edited the manuscript. Janice S. Blum edited the manuscript and oversaw its preparation.

## Conflict of Interest Statement

The authors declare that the research was conducted in the absence of any commercial or financial relationships that could be construed as a potential conflict of interest.
